# The current issues and future perspective of artificial intelligence for developing new treatment strategy in non-small cell lung cancer: harmonization of molecular cancer biology and artificial intelligence

**DOI:** 10.1186/s12935-021-02165-7

**Published:** 2021-08-26

**Authors:** Ichidai Tanaka, Taiki Furukawa, Masahiro Morise

**Affiliations:** 1 Department of Respiratory Medicine, Nagoya University Graduate School of Medicine, 65 Tsurumai-cho, Showa-ku, Nagoya, 466-8550 Japan; 2 Center for Healthcare Information Technology (C-HiT), Nagoya University, Nagoya, Japan

**Keywords:** Artificial intelligence, NSCLC

## Abstract

Comprehensive analysis of omics data, such as genome, transcriptome, proteome, metabolome, and interactome, is a crucial technique for elucidating the complex mechanism of cancer onset and progression. Recently, a variety of new findings have been reported based on multi-omics analysis in combination with various clinical information. However, integrated analysis of multi-omics data is extremely labor intensive, making the development of new analysis technology indispensable. Artificial intelligence (AI), which has been under development in recent years, is quickly becoming an effective approach to reduce the labor involved in analyzing large amounts of complex data and to obtain valuable information that is often overlooked in manual analysis and experiments. The use of AI, such as machine learning approaches and deep learning systems, allows for the efficient analysis of massive omics data combined with accurate clinical information and can lead to comprehensive predictive models that will be desirable for further developing individual treatment strategies of immunotherapy and molecular target therapy. Here, we aim to review the potential of AI in the integrated analysis of omics data and clinical information with a special focus on recent advances in the discovery of new biomarkers and the future direction of personalized medicine in non-small lung cancer.

## Introduction

To improve prognosis of cancer patients, there is a growing trend to analyze numerous types of omics data, such as DNA, RNA, microRNA, protein, and metabolites [1, 2]. Many researchers have been aiming to develop identified markers for clinical application of early cancer detection, prognosis prediction, and evaluation of treatment efficacy. The recent advent of next generation sequencing (NGS) has permitted the generation of comprehensive profiles of somatic mutations in various cancer types and has contributed to the rapid advancements made in the field of cancer research. Genome sequence-based studies of large numbers of clinical samples, such as those available through The Cancer Genome Atlas (TCGA) and International Cancer Genome Consortium (ICGA), have led to the identification of a variety of driver gene mutations and oncogenic signaling pathways that give cancer cells a fundamental growth advantage during their neoplastic transformation [3–7]. These studies have revealed significant genomic heterogeneity, not only in different regions of the same patient, but also within a single tumor, and are contributing to the elucidation of the essential qualities of cancer development and progression [8]. Furthermore, numerous biological networks of genetic mutations affecting DNA copy number, methylation, the proteome, and the transcriptome have been dramatically demonstrated in cancer systems [9–12]. In addition, the recent advanced omics-technologies allow us to conduct single cell multi-omics sequencing, which can characterize the unique genotype and phenotype of each individual cell. This approach can provide new insights into tumor heterogeneity and deep characterization of the tumor microenvironment at a single-cell resolution [13]. Therefore, integration of these diverse omics data with highly accurate clinical information should lead to new clinical developments regarding the prevention of cancer onset and new treatment strategies based on intratumoral heterogeneity.

In parallel with the development of omics data analysis, recent exploitation of artificial intelligence (AI)-based technology has progressed rapidly. The theory of AI itself has existed since around the time of World War II, but several endeavors at developing AI failed due to problems associated with the lack of computing power. However, the application of AI in molecular biology has become more common with the advancements in computer technology. In accordance with the development of AI technology, trained deep learning has gradually evolved and currently plays an important role in clinical applications, especially in analyzing radiographs [14–16] and pathological images [17–19]. Meanwhile, the machine learning approach remains to be used mainly for omics data analysis due to the features of small sample sizes and large dimension data [20]. Multiple omics databases, such as TCGA and ICGA, have been dramatically expanded. Additionally, recent new multilayer omics analyses, such as single-cell sequencing, have been generating an extremely huge amount of data, resulting in the rapid evaluation of these massive amounts of data being beyond the capabilities of manual analysis. To reduce the level of labor involved in analyzing huge amounts of complex omics data, successful collaborations between biologists and computer scientists are required. Ultimately, machine learning approaches will play a central role in the creation of efficient strategies for promoting positive cancer research outcomes.

Among the numerous types of cancer, lung cancer can be a pervasive disease that is commonly diagnosed at advanced stages, with non-small-cell lung cancer (NSCLC) being the most prevalent form of lung cancer [21]. In recent decades, two innovative treatment strategies have been established to achieve long-term survival of patients with advanced NSCLC. The first one was based on the discovery of druggable oncogenic driver mutations or fusions. The second was the development of immune oncology, which is especially represented by immune checkpoint inhibitors (ICIs). Pivotal clinical trials have led to the establishment of a variety of first-line therapies as standard treatment strategies for subgroups of patients with NSCLC based on oncogenic driver mutation status and programmed death-ligand 1 (PD-L1) tumor proportion scores [22–25]. Furthermore, various clinical trials investigating new compounds or combination therapies with existing antineoplastic agents have been performed or are ongoing for each subgroup of NSCLC. Unfortunately, primary and acquired resistance against new strategies are a relevant issue and a primary concern as resistance complicates the decision of choosing the best therapeutic strategy among the numerous treatment options available. Therefore, establishment of AI-based comprehensive predictive models for efficacy and toxicity of each treatment is particularly desirable in terms of further developing individual treatment strategies. In this review, we summarize recent medical applications of AI for the analysis of omics data in combination with clinical information for NSCLC and discuss future application of this magnificent and powerful technology to clinical fields.

## AI in medicine—concepts and utilization

### Classification of AI

According to the algorithm used, AI is categorized as “rule-based,” which is called AI in a broad sense, and “non-rule-based,” which is referred to as machine learning. For rule-based algorithms, a person provides conditional branches and rules to solve for an optimal answer. For example, if a person defines the AI algorithm with the condition that "when study number of two databases are the same, they are regarded as duplicates and should be integrated," the algorithm will be fully faithful to the command and integrate the numbers. A rule-based algorithm is effective in limited situations in which there are limited choices. However, it is difficult to create a rule-based algorithm under complicated situations.

In contrast, machine learning automatically generates rules from known training data and applies them to the machine-learning algorithm using statistical analysis. Therefore, machine learning is focused on reading patterns from a large amount of data in a short amount of time and can semi-automatically obtain more accurate results than that of manual human evaluations. Machine learning is classified into three types, supervised learning, unsupervised learning, and reinforcement learning [26, 27]. Supervised learning is a technique in which the learner parameter is updated in order to get closer to the correct output. In other words, training data are provided to the algorithm, the correct answer label is learned, and a learning algorithm is generated in which the output is the correct answer label. Next, it is verified whether a value close to the "correct label" is obtained when unknown data is applied to the generated model. This type of machine learning is usually used for classification tasks or regression tasks in image recognition. For example, when a whole slide image of lung cancer is provided as an input and the output is labeled as “normal lung”, the learner will be updated by the teacher that the correct answer is “lung cancer” [28]. Supervised learning requires an extensive amount of training data and the following labeled data, which are often difficult to obtain in medical and biological fields. Meanwhile, unsupervised learning is another machine learning technique in which the learner is updated using only inputs without “correct answer” data. Reinforcement learning is the final machine learning technique, and updates the learner through trial and error in order to determine the best course of action to suit the current situation.

Deep learning is a machine-learning technique inspired by the human brain that uses large mathematical functions with millions of parameters based on a neural network structure that combines multiple layers of artificial nerve cells [16]. Using a deep-learning system, great power can be exerted for the recognition and classification of various medical images and is applicable to pathological diagnosis and cancer detection using computed tomography (CT) images [15, 29–31]. While most of deep-learning algorithms to date have been applied using supervised-learning methods to learn a specialist’s thought or technique, some deep-learning algorithms have been recently created using unsupervised-learning methods. For instance, Yamamoto et al. developed a deep-learning algorithm that enables an automated acquisition of explainable features from diagnostic annotation-free histopathology images of prostate cancer and identified a new feature that improves the accuracy of diagnosis of prostate cancer recurrence [32]. Essentially, they created a deep-learning algorithm that uses histopathological images of prostate cancer as inputs, which then automatically outputs feature maps of the histopathological images.

### Application of AI for analysis of omics data and clinical information

**Fig. 1:**
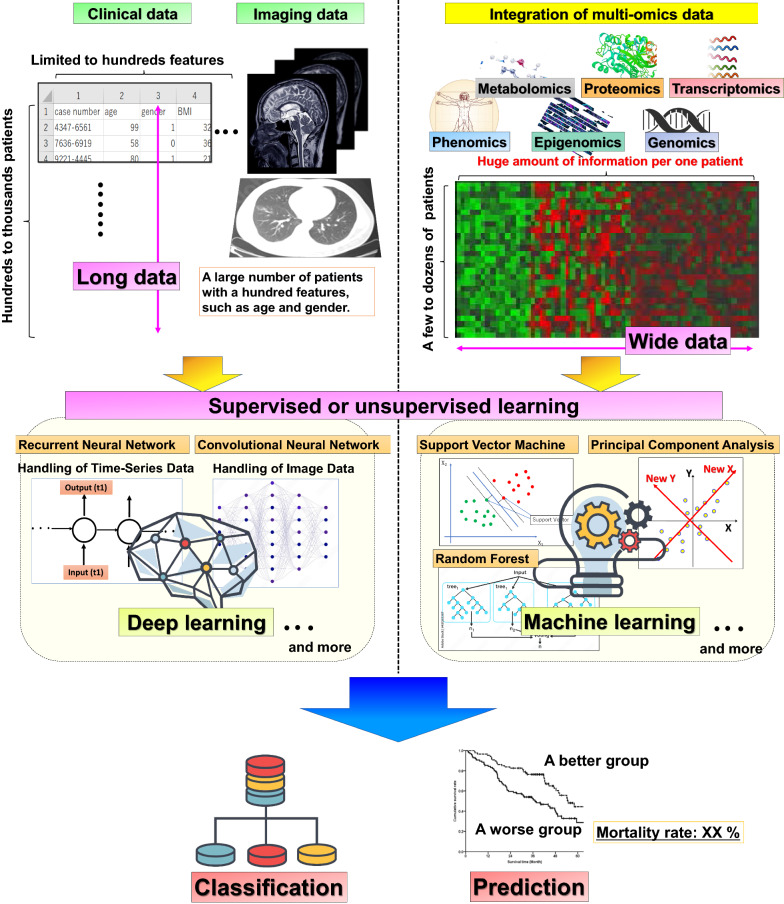
Application image of artificial intelligence use for analysis of omics data and clinical information

The application of AI in medicine is currently of great interest, especially in the diagnostic and predictive assessment of medical images [33]. Among AI algorithms, machine learning is able to learn health trajectory patterns from vast numbers of patients (Fig. 1). This can help physicians anticipate future events at an expert level and draw curves from extensive amounts of clinical information, providing insight well beyond the experience from an individual physician’s practice. Development of an algorithm for medical diagnosis or prediction typically requires a huge dataset, often referred to as “big data,” especially an algorithm in which supervised learning of deep neural networks are used. Accurate algorithms require high quality datasets; however, these big datasets need to be collected in various ways from multiple heterogeneous sources [34]. When the algorithm diagnosis outputs in the training phase differ from the actual diagnosis, the calculated parameter weights are updated in order for the output to approach the correct disease label. This process is then repeated many times. During the updating process, deep learning generally requires an extremely large number of samples to approach the correct answer as the algorithm parameter may exceed one hundred million.

Although the samples available for omics data analysis have been usually limited, deep-structured learning usually requires an extremely large number of samples. Therefore, machine-learning models have been commonly utilized to create quicker and more accurate algorithms under current situations for the analysis of omics data (Fig. 1). Because the inclusion of too much features may lead to overfitting and increases calculation costs, each analysis initially starts with biomarker selection and knowledge of omics data, as well as selection of statistical methods that increase the stability of the feature selection process. In biomedical analysis, the term “features” indicates measured characteristics used as learning input, such as age, gender, or X genes.

After feature selection, machine learning can be used to achieve various ends, such as disease type or severity classification or mortality prediction. To analyze omics data using machine learning, feature selection is one of the most important procedures because of its large dimension. Interestingly, machine-learning techniques have sometimes been used in the feature selection procedure itself [35]. Best et al. selected specific spliced-RNA biomarker panels using likelihood ratio analysis of variance (ANOVA) statistics and then comparing healthy individuals to patients with cancer based on analysis of differential expression of spliced junctions [36]. Logistic regression analysis, ANOVA statistics, and an ensemble approach with random sub-sampling have been widely used to select important features [37–39]. Another way to reduce the dimension of potential features is using unsupervised-machine learning, such as least absolute shrinkage and selection operator (LASSO) regression or principal component analysis (PCA) [40]. LASSO regression, one approach of regression analysis, has the feature of part of the coefficient being set to zero, reducing that dimension of the feature. Lu et al. obtained 2139 genetic mutations for consideration in their initial model to predict long‑term clinical benefit, which was then reduced using the LASSO model to 161 genetic mutations without a reduction in the clinical prediction accuracy [31]. Meanwhile, PCA weighs and integrates many features to create a relatively small number of new features that represent the overall variability. Guan et al. performed PCA to distinguish patients with inflammatory bowel disease (IBD) from control subjects using 55 lipid species [40]. They defined two new features of principal component 1(PC1) and principal component 2 (PC2), which were able to distinguish between patients with IBD and the control subjects. These filtering steps improve data normalization, which is a critical step in biological data analysis.

Generally, supervised and/or unsupervised learning models, including LASSO, support vector machine (SVM), random forest, and gradient boosting, have been used after feature selection to perform the classification task, such as identifying patients with significantly worse mortality rates. SVM is an algorithm that minimizes the distance of prediction error and is one of the most frequently used systems of supervised machine learning in omics data analysis. The advantage of SVM compared to that of other algorithms is its good accuracy and use of fewer parameters to be optimized, even if the data dimension is large. However, the disadvantages of the SVM algorithm are the large calculation costs as the amount of training data increases; therefore, feature standardization will be needed.

## Identification of early detection biomarkers in NSCLC using omics data and AI

### The application of AI in imaging diagnostics for NSCLC screening

Most patients with NSCLC have advanced stage disease with distant metastasis at the initial diagnosis. The five-year survival rate of patients diagnosed at stage IV NSCLC is only 6.0% for patients that receive historic cytotoxic chemotherapy regimens, while the five-year survival rate dramatically rise to around 70–90% for patients diagnosed with stage I NSCLC [41]. Therefore, early detection of NSCLC is extremely effective toward improving the survival rate of patients. In 2011, the National Lung Screening Trial (NLST) showed that low-dose CT (LD-CT) screening for lung cancer reduced the relative mortality by 20% [42]. The all-cause mortality rate was 6.7% lower in the LD-CT group compared to that in the X-ray group. The US Preventive Services Task Force recommends an annual LD-CT screening test for high-risk populations, which comprises patients with a smoking history of at least 30 pack-years and an age of 55 to 80 years. However, the inclusion criteria excluded young subjects and never-smoker or light-smoker populations. Furthermore, LD-CT screening is costly with high false positive rates because of the detection of benign pulmonary nodules [43].

To detect ever-smaller lung tumors and to improve the accuracy of CT screening, the development of AI-based screening methods for all populations is rapidly progressing. Currently, complex algorithms and various types of software devices have been utilized to develop AI-based screening methods, and these are mainly categorized into two systems, namely, computer-aided detection (CADe) system and computer-aided diagnosis (CADx) system [44]. The CADe system, which highlights the detection of small nodules, has been engineered to improve radiologist sensitivity in identifying nodules. The CADx platforms can support diagnosis of pre-identified lesions when clinicians evaluate malignancy risk or conduct clinical decision-making. The development of both the systems is indeed important for improving diagnosis correctness, early diagnosis, and reducing diagnostic variation owing to clinician’s subjectivity. However, most of the recent studies involve small sample sizes or no validated models, and therefore, AI-based screening methods are not enough for clinical application at this point of time.

To surmount the current difficulties, several frameworks of academia–industry collaboration have been gradually established worldwide. Optellum Ltd., a company that specializes in image analysis of lung cancer diagnosis, developed a machine leaning algorithm called the lung cancer prediction convolutional neural network (LCP-CNN), which was initially trained using the NLST data under guidance from experienced thoracic radiologists at Oxford University Hospitals [27, 45]. Subsequently, to compare the performance of LCP-CNN with that of the Brock University model, recommended by United Kingdom (UK) guidelines, Baldwin et al. conducted a validation study by retrospectively collecting data from 5–15 mm lung nodules, which consisted of 1187 patients with 1397 nodules from three hospitals in the UK [45]. In this study, the area under the curve (AUC) for LCP-CNN was 89.6% compared with 86.8% for the Brock model (p ≤ 0.005), resulting in a better discrimination ability of LCP-CNN with over 99.5% sensitivity compared with that of the Brock model. As another model of academia–industry collaboration, Ardila et al. used the TensorFlow platform of Google Inc., to develop a deep-learning model trained using 42,290 CT scan images from 14,851 patients, which is able to determine the malignancy of lung nodules without the need for human intervention [46]. The AI-equipped system detected minute malignant lung nodules in 6716 test cases with an accuracy of 94%. The model performed better than six radiologists who made the diagnosis in the absence of previous CT images [46]. The approach was undertaken in a collaboration between Google, Northwestern University, and other institutions, and is one of the systems moving toward clinical adoption. Both these studies showed successful academia–industry collaboration on radiomics and AI-based screening, which can make the detection of early lung cancer more precise and accessible for all the population.

### The application of omics data and AI for identification of early detection biomarkers

For the purpose of supplementing the false positivity of LD-CT screening or earlier detection of lung cancer than that achieved using CT, a variety of new technologies have been investigated over the past decade for the discovery of biomarkers from various biomolecules. For example, because of dramatic advances in the accuracy of mass determination and characterization of target proteins, mass spectrometry (MS) has been developed to analyze a diverse range of proteins, lipids, and metabolites. Currently, it is possible to detect extremely small amounts of protein from tiny cancers using the advanced MS technology. In addition, Taguchi et al. performed proteomics analysis using blood samples obtained from various cancer mouse models and found levels of the N-terminal pro-peptide of surfactant protein B (pro-SFTPB) are characteristically increased in the blood of mice with lung cancer [47]. Diagnostic blood tests, including for pro-SFTPB, may be able to identify people with lung cancer up to 2 years earlier with about twice the sensitivity of the current LD-CT criteria [48–50]. This indicates the combination of LD-CT screening and detection of protein-based biomarkers will be truly effective for the accurate and early detection of lung cancer.

The use of machine-learning approaches and the accumulation of omics data in recent years have led to more sensitive and accurate detection of biomarkers. For instance, Noreldeen et al. described a non-targeted lipidomic approach based on ultra-high-performance liquid chromatography coupled with quadrupole time-of-flight MS in combination with two machine learning approaches (genetic algorithm and binary logistic regression) to screen candidate discriminating lipids and to define a combinational lipid biomarker in serum samples for distinguishing female patients with NSCLC [51]. They showed that fatty acid (FA) (20:4), FA (22:0), and lysophosphatidylethanolamine (20:4) can serve as a combinational biomarker for distinguishing female patients with early-stage NSCLC from healthy controls with good sensitivity and specificity and the AUC reaching 0.99. In a study using machine learning to parse omics data other than protein-based data, Best et al. analyzed RNA biomarker panels from platelet-derived RNA-sequencing libraries using particle-swarm optimization (PSO)-enhanced algorithms [52]. Their results showed accurate tumor-educated blood platelets (TEP)-based detection of early-stage NSCLC (AUC, 0.89). Because the characteristics AI are from a completely different perspectives than that of earlier reports, the AI studies may lead to the elucidation of molecular biological mechanisms of lung cancer progression, as well as the identification of biomarkers for its early detection. It is expected that AI in the future will be able to integrate diagnostic imaging with new biomarkers using the comprehensive analysis of omics data.

## Development of immune checkpoint inhibitors (ICIs) treatment of NSCLC based on AI analysis of OMICS data

### Current issues in the standard treatment strategy of NSCLC and further development of immune therapy

**Fig. 2:**
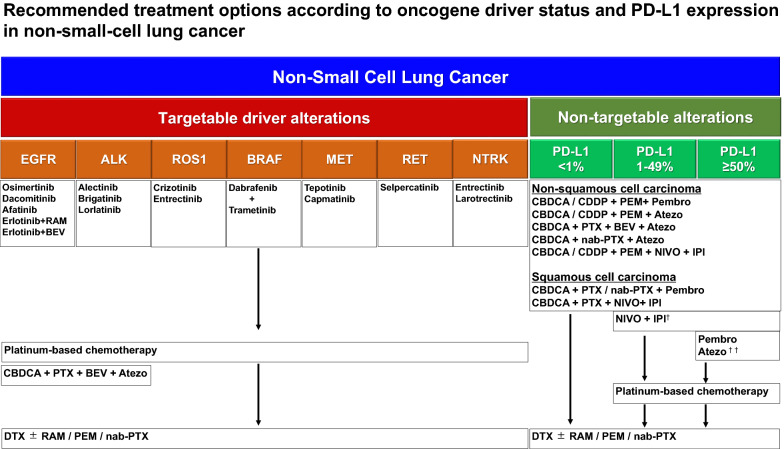
Recommended treatment options according to oncogene driver status and PD-L1 expression in non-small-cell lung cancer. Next generation tyrosine kinase inhibitors for each driver oncogenic aberrations are approved one after another, and novel immune check point inhibitors combination with or without platinum-based chemotherapy are established as standard treatment options for non-small cell lung cancer without druggable alterations. ^†^Nivolumab plus ipilimumab is an option for patients with PD-L1 tumor proportion score < 1% in addition to those with PD-L1 tumor proportion score ≥ 1%. ^††^High PD-L1 expression is defined as ≥ 50% of tumor cells or ≥ 10% of tumor-infiltrating immune cells by SP142 assay. CBDCA: Carboplatin; CDDP: Cisplatin; PTX: Paclitaxel; DTX: Docetaxel; PEM: Pemetrexed; nab-PTX: nanoparticle albumin bound Paclitaxel; BEV: bevacizumab; RAM: Ramucirumab; NIVO: Nivolumab; IPI: ipilimumab; Atezo: atezolizumab; Pembro: pembrolizumab

Pivotal phase III clinical trials have led to the worldwide approval of ICIs, such as programmed cell death 1 (PD-1)/programmed death-ligand 1 (PD-L1) inhibitors and cytotoxic T-lymphocyte-associated protein 4 (CTLA-4) inhibitors, especially for non-squamous NSCLC without sensitizing *Epidermal Growth Factor Receptor* (*EGFR*) mutation or *anaplastic lymphoma kinase* (*ALK*) fusion, and squamous cell lung carcinoma [53–58]. Multiple treatment regimens, including PD-1/PD-L1 inhibitors with CTLA-4 inhibitors and PD-1/PD-L1 inhibitors with or without CTLA-4 plus platinum-based chemotherapy, are currently standard treatment options (Fig. 2). However, the optimal regimen for individual patients remains unclear as these new treatment regimens were compared to traditional platinum-based chemotherapy as the control-arm in all the phase III studies. Furthermore, comparative clinical trials of these new regimens have not been conducted and “round robin” clinical trials comparing these regimens may be unrealistic. However, novel immune therapeutic agents other than PD-1/PD-L1 inhibitors and CTLA-4 inhibitors are currently being investigated in several clinical trial settings [59], suggesting multiple combination therapies of immune targeting drugs may be approved before long as additional standard treatment options. Accordingly, the development of patient selection strategies for individualized immunotherapy is an important issue. In recent decades, comprehensive analyses of tumor specimens combined with detailed clinical information have been performed in various clinical trials [60]. Although these large profiling datasets have the potential to benefit the discovery of novel prediction methods of immune therapeutic activity for individual patients, translational and reverse translational research has not been adequately conducted. Under these circumstances, machine-learning approaches are some of the most promising technologies for identifying new biomarkers from various omics data that can be used to drive individualized immunotherapy.

The evaluation of PD-L1 expression and tumor mutation burden (TMB) using immunohistochemistry have been widely adopted as markers for ICI treatment [61, 62]. However, the predictive ability is insufficient to adequately stratify patients for proper treatments compared to that of targeted oncogenic driver aberrations. These limitations are associated with immune responses being affected by tumor-cell specific features of NSCLC, immune-cell specific features, and the tumor microenvironment [63]. Using AI to establish a comprehensive prediction model for immunoblockade strategies will result in relevant advantages compared to that of traditional biomarker analysis. Once AI technology is able to identify populations with innate resistance to specific standard ICI regimens based on large clinical datasets of comprehensive tumor samples from clinical trials or real-world data (RWD), such populations will be a good target for further clinical trials of new ICI combination therapy aimed at overcoming innate resistance to ICI regimens. In addition to selecting suitable target populations for clinical trial settings, standard machine-learning approaches are expected to identify relevant biomarkers and clinical factors as each variable is interpretable using machine-learning methods. Furthermore, it will be possible to conduct subsequent reverse-translational research based on AI-driven interpretable biomarker profiling to determine the biological mechanism of primary resistance to specific standard ICI regimens. The harmonization between biological approaches and AI technology, supported by basic biological rationale, should foster the next generation of clinical trials with improved probability of positive clinical trial results.

### Prognostic biomarker of ICI treatments using omics and AI

Much of the accumulated evidence regarding the relationship between specific driver gene mutations and the immune microenvironment is based on recent NGS analyses. Two tumor suppressor genes in NSCLC, *serine/threonine kinase 11* (*STK11*) and *Kelch-like ECH-associated protein 1* (*KEAP1*), are widely known as representative inactivated mutations with immunosuppressed phenotypes, regardless of PD-L1 expression and TMB [64–66].

Liver kinase B1 (LKB1), which is encoded by *STK11*, regulates cell polarity and functions as a tumor suppressor with germline mutations in this gene being related to the autosomal dominant disorder Peutz–Jeghers syndrome [67]. LKB1 inactivation is detected in approximately 20% of lung adenocarcinomas, effects tumor initiation, and uniquely confers invasive and metastatic properties through the reprograming of energy metabolism, such as glucose/FA uptake and pyrimidine/purine balance [68–71]. In the NSCLC tumor microenvironment, LKB1 inactivation is shown to downregulate PD-L1 expression and promote proinflammatory cytokine production to suppress T-cell infiltration [72].

Meanwhile, KEAP1 is an adaptor for a cullin-3 (CUL3)-based ubiquitin ligase and is involved in the control of oxidative stress to facilitate ubiquitination and the subsequent proteolysis of nuclear factor erythroid 2-related factor 2 (NRF2), which is a master regulator of the antioxidant response. Loss of KEAP1 or CUL3 function results in constant NRF2 activation and the tumors exhibit resistance to radiotherapy and cytotoxic chemotherapy [73–75]. NSCLC with LKB1 inactivation and/or disruption of the NRF2-KEAP1-CUL3 complex are widely known to demonstrate an aggressive clinical course, shorter survival rates, and resistance to ICIs treatments. Furthermore, recent multi-omics analysis has determined that activating mutations in receptor tyrosine kinases genes, such as *EGFR* mutations, *human epidermal growth factor receptor 2* (*HER2*) point mutations and amplifications, *MET Proto-Oncogene, Receptor Tyrosine Kinase* (*MET*) amplification, *fibroblast growth factor receptor 1* (*FGFR1*) amplification, and *insulin like growth factor 1 receptor* (*IGF1R*) amplification, are linked to primary resistance to ICIs, independent of PD-L1 expression and TMB [76]. Among these, EGFR activation shows various immunosuppressive mechanisms to suppress tumor-infiltrating lymphocytes, including the expression of CD73 and secretion of T-cell inhibitory molecules [77–80]. Conversely, several driver gene mutations, including *AT-rich interactive domain-containing protein 1A* (*ARID1A*), *Janus kinase 1* (*JAK1*), and *Janus kinase 2* (*JAK2*) mutations and co-occurring *KRAS* mutations and *TP53* inactivation, are associated with T-cell infiltration and reflect favorable responses to ICIs therapies with high expression of tumor antigens [66, 76, 81, 82]. Therefore, the widespread utilization of NGS-based testing, which is currently tending to decline in cost, will help guide the selection of good responders to ICIs.

Other monolayer omics analyses have also led to the elucidation of the immune-microenvironment of individual tumors and to the establishment of predictors for ICIs therapeutic efficacy. For instance, examination of whole-exome signatures of mutagenic biological processes within tumor specimens has found an enrichment of the C > A transversion-rich molecular tobacco-smoking signature in patients with durable benefits by ICIs treatment [83]. When a tobacco-smoking signature is detected, the total number of single-base substitutions is shown to associate with TMB and more accurately predicts ICIs response than TMB [76]. Tumor-specific neo-peptides linked to T-cell infiltrates in tumors and the clinical efficacy of ICIs have also been well investigated in various cancer types [84, 85]. For effective tumor killing, CD8+ T cells must recognize the neo-peptides presented by human leukocyte antigen class I (HLA-I) molecules. Deficiency of antigen presentation is associated with immune escape through both HLA class I germline homozygosity and the loss of heterozygosity, which then influences the response of cancer to ICIs [86, 87]. However, these monolayer omics analyses may be less effective in accurately predicting the outcome of treatment with ICIs and multimodal approaches might be needed [76].

In an effort to accurately classify patients with ICI response, Lu et al. attempted to establish a proper model using machine learning and whole-exome sequencing data [37]. They used metastatic melanoma as training data and validation was conducted using a NSCLC dataset. From the initial model, which considered 2139 mutations, their machine learning technique selected 161 mutations (11%). In the NSCLC cohort, the high-weight-TMB group was found to be associated with better survival and better 6-month clinical benefit was predicted (AUC = 0.83). Interestingly, among the 161 mutations, only nine genes (< 6%) had negative coefficients and the weighted gene mutation selected by their machine-learning technique was consistent with previous mutation load markers based on molecular omics analysis.

Meanwhile, Wiesweg et al. conducted machine learning approaches on RNA expression of a 770-gene panel covering immune-related genes in patients with advanced NSCLC, in combination with PD-L1 immunohistochemistry [39]. The model prediction plus PD-L1 positivity identified NSCLC patients with highly favorable outcomes.

In addition to NGS analysis, integrated analysis based on multi-omics data including tumor-adjacent tissue, should allow for construction of new models for the accurate prediction of therapeutic efficacy. In addition to the application of machine learning for omics data analysis, several studies have developed deep learning to predict ICIs efficacies using pathological images and clinical information. For instance, Khalid et al. conducted integrative analysis of spatial histological images by training deep-learning algorithms in addition to analysis of multi-region exome and RNA-sequencing data in 100 patients with NSCLC [88]. The study demonstrated that lung adenocarcinomas with more than one immune-cold region were at significantly higher risk of cancer relapse, regardless of the number of total regions sampled and the immune phenotypes of the other regions. In this way, AI-based analysis using omics data and clinical information can provide a completely new perspective on predicting therapeutic effects. As the next research strategy, integration of multilayer omics data with machine-learning analysis in combination with analysis of clinical information, such as CT and/or histopathological images, by training deep-learning will provide currently insensible prediction models.

## Future direction and challenges of using AI in NSCLC with druggable mutations

### Current issues of molecular targeted drug discovery and clinical trials in NSCLC with oncogenic driver aberrations

Several oncogenic driver mutations and oncogenic fusions have been established as therapeutic targets for NSCLC. In such oncogenic driver aberrations of NSCLC, *EGFR*, *ALK*, *MET*, and *B-Raf proto-oncogene serine/threonine kinase* (*BRAF*) mutations, and *ALK*, *ROS proto-oncogene 1 receptor tyrosine kinase* (*ROS1*), *ret proto-oncogene* (*RET*), and *neurotrophic receptor tyrosine kinase* (*NTRK*) fusions have been identified and the clinical benefit of several tyrosine kinase inhibitors (TKI) targeting these oncogenic driver mutations and fusions have been proven by well-designed clinical trials (Fig. 2) [41]. Targeted therapy of oncogenic driver mutations and oncogenic fusions in NSCLC achieve higher response rates with longer duration of progression free survival (PFS) compared to conventional cytotoxic agents. However, several issues remain in the further development of individualized treatment strategies for oncogenic driver mutations and fusions. For instance, the clinical benefit for each oncogenic driver aberrations depends on both the inhibitory ability of a specific targeted oncogenic driver aberration and tolerability. Thus, the discovery of new compounds that exhibit highly selective inhibitory effect for targeted oncogenic driver aberrations is one of the most crucial steps in the development of new standard treatments.

As an example, we review the history of developing an *ALK*-fusion targeted therapy. The discovery of ALK dates back to 1994 when a chromosomal rearrangement, t(2;5), resulting in a *nucleophosmin* (*NPM1*)–*ALK* fusion was described in anaplastic large-cell lymphoma [89]. More than a decade later, subsequent work identified the *ALK* fusion proteins as oncogenic driver alterations in a variety of cancer types. Among them, the *echinoderm microtubule-associated protein-like 4* (*EML4*)–*ALK* fusion was recognized in 2007 as a representative oncogenic driver fusion in approximately 3–7% of NSCLC [90]. Several years later, the first approved agent for *ALK* fusion, crizotinib, was shown to exhibit superiority over cytotoxic chemotherapy [91, 92]. However, because of its inhibitory activity on several tyrosine kinases in addition to ALK, such as ROS1 and MET among others, crizotinib frequently causes various adverse events (AEs), including nausea, bradycardia and transient visual disorders [93]. The severe AEs sometimes lead to the targeted therapy being discontinued. Therefore, the development of a new agent with high selectivity for oncogenic *ALK*-fusion signaling was necessary as a next step to achieve further long-term tumor control with less toxicity. The second generation ALK-TKI alectinib was designed to inhibit the ALK tyrosine kinase with high selectivity [94]. Based on the results of three phase III clinical trials that proved the superiority of alectinib with PFS as the primary endpoint over that of crizotinib with less toxicity, alectinib was approved in 2017 as a first-line standard agent [95–97]. This developmental history of a molecular targeted therapy is one of the success stories for the patients with oncogenic driver aberrations; however, drug discovery and developments starting from traditional screening methods to clinical trials is an extremely expensive and time-consuming procedure. Moreover, as another major issue, less than 10% of agents entering clinical trial settings achieve successful results and the Food and Drug Administration approval [98]. Furthermore, approximately 20% of tumors show innate resistance and early tumor progression based on several biological characteristics, such as intratumoral heterogeneity and other driver mutations. The remaining tumors subsequently acquire resistance through various molecular mechanisms, including secondary mutation of the same driver gene or activation of other oncogenic signals. Most oncogenic driver aberrations are themselves only a relatively rare fraction of the tumor cell population. The subpopulation classified by a specific resistant mechanism of each oncogenic driver aberrations is increased through the variety of aberrations [99]. Therefore, screening promising new targeted strategies for overcoming resistance mechanisms determined by oncogenic aberrations in each specific subpopulation and conducting multiple phase I/II trials based on traditional methods seems unrealistic.

### Potential role of AI in development of new treatment strategies targeting oncogenic driver aberrations

**Fig. 3:**
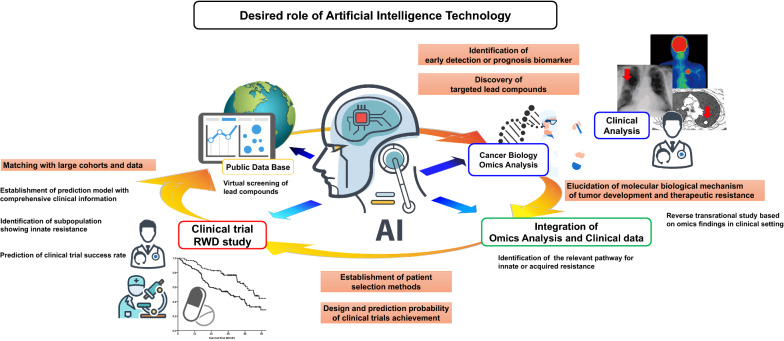
Future direction and potential role of artificial intelligence for development of new treatment strategies

The discovery of highly selective inhibitors that target oncogenic driver aberrations is a crucial step in the ultimate approval of a novel standard molecular targeted therapy. AI is expected to play several roles in the development of new treatment strategies (Fig. 3). First, AI enables the virtual screening of targeted lead compounds using multiple public databases, such as TCGA, the Human Protein Atlas and DrugBank, and PubChem. AI-based virtual screening supports the identification of candidate compounds with highly specific selectivity for targeted oncogenic driver aberrations and low toxicity. For example, Istvan et al. reported an AI-assisted computational method, which is a proprietary technology of Oncompass Medicine Inc., to prioritize potential molecular targeted therapies based on the complex individual molecular profile of the tumor in each patient [100]. They analyzed the clinical benefits of the digital drug-assignment system using the data from the SHIVA01 precision oncology clinical trial, and showed that the system identified substantial molecular targets with the fitting inhibitors, including in lung cancer patients, such as *FMS Related Receptor Tyrosine Kinase 3* mutation with sorafenib and *Androgen receptor* expression with abiraterone. These findings indicate that the AI-assisted computational systems for prioritization of potential molecular targeted therapies would be promising to improve the clinical benefits of precision oncology. As another example, in recent studies, it has been reported that the discovery of selective heat shock protein 90 inhibitors and an aurora A inhibitor was driven by virtual screening [101, 102]. These ligand-based virtual screening methods will be a powerful tool in selecting a new and ideal inhibitor against previously identified molecular targets. Because resistance to targeted drugs of oncogenic driver aberrations can emerge through secondary oncogenic driver aberrations that exhibit various molecular mechanism of resistance, the virtual screening of compounds can promote the cost-effective development of treatment strategies for overcoming heterogeneous mechanism of resistance. Traditional screening methods combined with conducting multiple phase I/II trials are currently required to replenish the pool of potential innovative development strategies and new drugs for targeting oncogenic driver aberrations. In addition to the discovery of targeted compounds, AI will be contributing on the prediction of success rates of clinical trials. Indeed, Gayvert et al. reported that a new data-driven approach is able to predict clinical toxicity and may identify compounds in clinical trials with acceptable toxicity [102]. Improving the probability of success for clinical trials based on AI would also help resolve the current issue of a limited availability of patients with rare oncogenic driver aberrations.

## Prospects

Precision medicine in the treatment of lung cancer has shown a dramatic growth with progression in harmonization of molecular cancer biology and AI-based technology. AI, radiomics, and molecular cancer biology exhibit mutual influence, and can generate powerful AI systems for further development of individual treatment strategies. Although accumulating recent omics data have consecutively provided a variety of new biological insights, the analysis may have been beyond the capabilities of manual analysis. Therefore, establishing new framework for analyzing huge size of omics data, such as academia–industry collaboration and academia–government technological collaboration, will be important as well as the AI development. With regard to radiology and molecular targeted therapies, some academia–industry collaborations have successfully complemented each other [45, 46, 100], and AI-based screening has been accelerated toward clinical applications. Prospectively, these frameworks, which can lead to further progression of inter-industry activities, and medical AI systems could be a detector of microchanges in patients that can go unnoticed by human eyes, and be a selector of suitable treatments for individual patients to support clinicians, resulting in more early intervention and in improving the quality of life of patients.

Moreover, comprehensive profiles of individual omics data are increasingly important not only to patients but also to their families and blood relatives. Additionally, recent AI-based systems for multi-omics analyses have an increased possibility of accidental and unexpected discoveries to affect an individual’s life. Patients should have the opportunity to know how their data is being shared and used, and the enormous individual data should be protected against risks of disclosure. Therefore, omics information holders have ethical and legal obligations, big responsibilities for data stewardship, and regulatory issues for decision-making. Under the current law structure, these ethical and legal issues may not be satisfactorily served with regard to various aspects including those in the area of intellectual property. In parallel with the rapid development of AI-based omics data analysis, revisions to the legal framework would also be needed.

## Conclusions

Machine-learning and deep-learning technologies have undergone relevant advances, enabling the analysis large omics datasets and clinical information. Toward improving the prognosis of patients with NSCLC, AI has shown breakthroughs in potentially resolving current issues in the development of new treatment strategies, including for ICIs and molecular targeted therapy. These include, (1) identification of early detection or prognosis biomarkers, (2) elucidation of molecular biological mechanisms of tumor development and therapeutic resistance, (3) establishment of patient selection and stratification methods, (4) discovery of lead targeted compounds, and (5) design of clinical trials and prediction of their probable achievements or outcomes. In the coming decade, researchers will need to select suitable AI algorithms for analyzing expansive amounts of omics data and clinical information. Harmonization of molecular cancer biology and AI technology will dramatically improve research strategies and accelerate the creation of efficient outcomes that are beyond simply human capability.

## Data Availability

Yes.
